# Supplementary material for: Can the water resources tax policy effectively stimulate the water saving behavior of social water users? A DSGE model embedded in water resources tax
Source: PLoS One. 2023 Mar 10;18(3):e0281920. doi: 10.1371/journal.pone.0281920 (PMC10004565; doi:10.1371/journal.pone.0281920)
Supplement: Attachment — Submitted filename: PLos One.docx [file pone.0281920.s001.docx]

**Title: Can the water resources tax policy effectively stimulate the water saving behavior of social water users?**

**Manuscript Number PONE-D-22-20549**

The manuscript entitled Can the water resources tax policy effectively stimulate the water saving behavior of social water users? is a good contribution to the existing body of knowledge in the related discipline. The paper used dynamic stochastic general equilibrium model (DSGE) embedded in water resources tax to simulate the persistent impact of water resources tax on water saving objectives.

- I found very poor English and sentence structuring throughout the paper. Hence extensive English editing may be required.

1. For example, use of “and” in the first line of abstract more than thrice.
2. Extraordinary long sentences misleading the true meaning of what the authors are trying to explain.
3. Study aim / need of study should be clear in the abstract
4. The objectives of the study are not narrated in proper English
5. It is good if the abstract become a bit short rather such long as the reader may get bored.

- Keywords may be changed
- The theoretical framework and the methodology are the most important and well elaborative part of the study and I want to appreciate the author for such deep insight
- I think there is a lack of references in the discussion section.
- All the results should be balanced with some references with prior studies and may be discussed in the discussion section.
